# Optimising Recycling Processes for Polyimine-Based Vitrimer Carbon Fibre-Reinforced Composites: A Comparative Study on Reinforcement Recovery and Material Properties

**DOI:** 10.3390/ma17102372

**Published:** 2024-05-15

**Authors:** Ákos Pomázi, Dániel István Poór, Norbert Geier, Andrea Toldy

**Affiliations:** 1 Department of Polymer Engineering, Faculty of Mechanical Engineering, Budapest University of Technology and Economics, Műegyetem rkp. 3, H-1111 Budapest, Hungary; pomazia@pt.bme.hu; 2 MTA-BME Lendület Sustainable Polymers Research Group, Műegyetem rkp. 3, H-1111 Budapest, Hungary; poor.daniel@gpk.bme.hu; 3 HUN-REN–BME Research Group for Composite Science and Technology, Műegyetem rkp. 3, H-1111 Budapest, Hungary; 4 Department of Manufacturing Science and Engineering, Faculty of Mechanical Engineering, Budapest University of Technology and Economics, Műegyetem rkp. 3, H-1111 Budapest, Hungary; geier.norbert@gpk.bme.hu

**Keywords:** vitrimer, carbon fibre-reinforced composite, chemical recycling, recycled carbon fibres

## Abstract

We investigated the recycling process of carbon fibre-reinforced polyimine vitrimer composites and compared composites made from virgin and recycled fibres. The vitrimer matrix consisted of a two-component polyimine-type vitrimer system, and as reinforcing materials, we used nonwoven felt and unidirectional carbon fibre. Various diethylenetriamine (DETA) and xylene solvent ratios were examined to find the optimal dissolution conditions. The 20:80 DETA–xylene ratio provided efficient dissolution, and the elevated temperature (80 °C) significantly accelerated the process. Scaling up to larger composite structures was demonstrated. Scanning electron microscopy (SEM) confirmed effective matrix removal, with minimal residue on carbon fibre surfaces and good adhesion in recycled composites. The recycled nonwoven composite exhibited a decreased glass transition temperature due to the residual solvents in the matrix, while the UD composite showed a slight increase. Dynamic mechanical analysis on the recycled composite showed an increased storage modulus for nonwoven composites at room temperature and greater resistance to deformation at elevated temperatures for the UD composites. Interlaminar shear tests indicated slightly reduced adhesion strength in the reprocessed composites. Overall, this study demonstrates the feasibility of recycling vitrimer composites, emphasising the need for further optimisation to ensure environmental and economic sustainability while mitigating residual solvent and matrix effects.

## 1. Introduction

Polymers play a pivotal role in creating a more sustainable world by offering versatile solutions across industries, from lightweight and durable materials in transportation to efficient packaging and renewable energy technologies, driving innovations towards a circular economy. Their unique properties, such as their lightweight nature, durability, and adaptability, enable polymers to replace traditional materials like metal and glass, reducing resource consumption, energy use, and emissions throughout their lifecycle. However, the integration of polymers into the circular economy, especially their recycling processes, still presents several challenges. The transition towards a circular economy for polymers requires not only the mitigation of their environmental impact but also fundamentally altering their design approaches [1]. While the mechanical recycling of thermoplastic polymers through melting and reprocessing is well established, developing recyclable crosslinked polymers remains challenging [2]. Thermosetting polymers, characterised by irreversible crosslinking, require different production, processing, and recycling techniques. Recycling thermosetting polymers has become inevitable despite their usage being less common compared to thermoplastics, owing to their increasing application, higher costs, and the demand for carbon fibres. Various recycling methods, including mechanical, thermal, and chemical processes, have been developed [3], but among these, only mechanical recycling and pyrolysis are widely adopted on an industrial scale. On the other hand, innovative concepts aim to create recyclable-by-design thermoset polymers and composites. In 2011, Leibler et al. [4] introduced vitrimers, a novel class of thermosetting materials based on associative covalent adaptable networks (CANs). Below their vitrimeric transition temperature (*T_v_*), vitrimers exhibit conventional crosslinked behaviour with excellent mechanical and thermal properties but can be melted and recycled above this temperature. Covalent adaptable networks (CANs) contain reversible covalent bonds that enable chemical responses to external stimuli [5]. These networks are classified into dissociative and associative CANs based on their bond exchange mechanisms [6]. In dissociative CANs such as polymers involving Diels–Alder reaction between furans and maleimides, bond-breaking precedes regeneration, compromising dimensional stability. Conversely, associative CANs like vitrimers maintain crosslink density throughout bond exchange [6]. Various bond exchange mechanisms, including transesterification, imine exchange, and disulphide exchange, have been explored for CANs [7,8,9,10,11,12]. Most of these bond exchange mechanisms are activated by specific catalysts, which imposes constraints on their application and impedes their widespread adoption [13]. Catalyst-free solutions such as polyimines synthesised from aldehydes or ketones and primary amines have been investigated due to their facile heat-triggered imine exchange reactions that also enable their chemical recycling through hydrolysis or transamination reactions [14,15].

Zhao et al. [16] synthesised a polyimine-based epoxy vitrimer by reacting vanillin with aminophenol, followed by glycidylation and consequent crosslinking with a commercially available polyfunctional amine. This vitrimer exhibited properties similar to traditional bisphenol-A-based thermosets but showed lower onset degradation temperature due to the increased dissociating tendency of imine bonds at elevated temperatures. The study demonstrated the recycling of imine-based vitrimers via a dissociative mechanism involving the acidic hydrolysis of imine linkages by hydrochloric acid solutions. The water was found to be the most efficient recycling solvent, followed by dimethyl sulfoxide (DMSO), dimethylformamide (DMF), ethanol, tetrahydrofuran, and benzene ≈ toluene, respectively, which was consistent with the polarity of the solvents. The recycling process involved dissolving vitrimer in acidified solvents, consequent drying to remove the solvents, and re-crosslinking at elevated temperature to recover the thermoset. The recycled thermoset retained a similar chemical structure and properties to the original thermoset, with no major decrease in tensile or thermal properties observed. However, challenges arose with solvent removal, especially for DMSO, which has a high boiling point and may impact the properties of the recycled thermoset.

Wang et al. [17] also synthesised vanillin-based polyimine-type epoxy vitrimers with different di- and triamines. Except for the vitrimer cured with diaminodiphenylmethane (DDM), all vitrimers were hydrolysed in 0.1 M HCl/DMF solution at room temperature for 2 h. The authors pointed out that the acid hydrolysis of epoxy vitrimers presented challenges in obtaining recyclable films, as the process resulted in the loss of hydrochloride amine, leading to an imbalance in aldehyde and amine groups. Despite attempts to regenerate films through acid solution evaporation, the presence of acid residues caused the film to hydrolyse easily to a liquid state. Therefore, they also investigated closed-loop recycling by transamination by adding excess isophoronediamine (IPDA) at 60 °C. The obtained amine-functional epoxy oligomers were then reacted with epoxy monomers. The recycled epoxy vitrimers showed a 17.2 °C lower glass transition temperature and inferior mechanical properties (1.67 ± 0.13 GPa and 32.3 ± 4.0 MPa) compared with virgin vitrimers. They also demonstrated the recycling of carbon fibre-reinforced composites with the hydrolysis method; however, the recycled carbon fibres were only characterised by scanning electron microscopy and Raman spectrometry, and the mechanical properties of the recycled fibres incorporated in composites were not investigated.

Memon et al. [18] synthesised an epoxy resin with dynamic imine bonds from lignin-derived vanillin and methylcyclohexanediamine (HTDA). Chemical recycling of the epoxy vitrimer was achieved via two methods. One method involved the degradation of epoxy networks through imine metathesis using ethylenediamine (EDA) at 100 °C, followed by recovery through EDA evaporation. The epoxy powders obtained after EDA removal were hot-pressed into new epoxy resins. The other method utilised HTDA for epoxy resin degradation, with the subsequent re-use of the obtained amine-functional oligomers for epoxy resin production by adding vanillin and epoxy monomers. The recycled resin from the second method retained 95% of the tensile strength of the pristine resin, whereas the first method yielded a recycled resin with only 74% of the original tensile strength. This reduction is likely attributed to residual EDA or potential defects introduced during the hot-pressing process. Both chemically recycled epoxy resins exhibited glass transition temperatures comparable to the pristine epoxy resin, suggesting no significant crosslinking density changes. In the carbon fibre-reinforced composites made from this epoxy vitrimer system, the matrix could be fully dissolved in EDA through amine–imine exchange reactions. After washing with ethanol and drying, they obtained soft reclaimed carbon fibre fabrics with no visible epoxy residues and fibre damage.

Xu et al. [19] developed a chemically degradable bio-based vitrimer from tung oil-based triglycidyl ester and menthane diamine. The carbon fibre-reinforced composites based on this imine-based vitrimer system exhibited excellent reprocessing, self-adhesive, and shape memory properties and could be rapidly chemically degraded by ethanolamine at 90 °C. The surface morphology, chemical structure, and tensile properties of the recycled carbon fibres were similar to those of the virgin fibres.

Liu et al. [20] developed a sustainable and high-performance epoxy vitrimer based on dynamic Schiff base chemistry using epoxidised soybean oil, vanillin, and 4-aminophenol. They recovered the carbon fibre reinforcements from the vitrimer matrix through acid-induced dissociation in a 0.1 M HCl DMF solution at room temperature. After 20 h, the matrix fully dissolved. The morphology and stress–strain curve of the recycled carbon fibre monofilaments closely resembled those of the originals, demonstrating that the composite could be recycled without destroying the fibre structure and compromising mechanical properties. Additionally, they conducted mechanical recycling of the composites through crushing followed by compression moulding. While the mechanical properties were noticeably diminished due to the shortened length of the carbon fibres, the reprocessed composite could potentially be utilised in other non-structural materials.

Taynton et al. [21] prepared polyimine vitrimers from terephthaldehyde and amines with different chain lengths and functionalities and tris(2-aminoethyl)amine (TREN). They demonstrated the chemical recycling of polyimine networks in carbon fibre-reinforced composites by introducing an excess of free primary amine groups (e.g., excess diamine monomer). Transimination reactions among the excess diamine monomers and the imine-linked network increased end groups within the matrix, thereby reducing the molecular mass and solubilising the network. The simple immersion of CFRC samples in neat diethylenetriamine (DETA) led to the complete dissolution of the polyimine vitrimer and the recovery of carbon fibre reinforcement.

Based on the literature, the chemical recycling of vitrimer composites is feasible; however, only a few studies address the conditions of chemical recycling and the mechanical properties of the recycled composites. In this research work, our goal was to investigate the effect of different parameters on the chemical recycling of imine-based vitrimers and the properties of recycled reinforcing fibres and composites produced from them.

## 2. Materials and Methods

In this study, carbon fibre-reinforced polymer (CFRP) composites were fabricated. A polyimine-type vitrimer system [22,23] was applied (called VITRIMAX T130) as a matrix material, and it was acquired from the supplier Mallinda Inc. (Denver, CO, USA). The two-component system consists of an epoxy resin part and an amine functional polyimine hardener part; the combined system’s viscosity is 54.8 Pa·s at 50 °C. The mixing ratio of the imine and epoxy was 2.5:1.

Two types of carbon reinforcements were used for the composite samples. For the preliminary investigation of the optimal recycling process, a nonwoven carbon felt was used (OX FT060-60 consisting of oxidised PAN fibres with an areal weight of 200 g/m^2^, supplied by Zoltek Ltd., Nyergesújfalu, Hungary). The other type of composite samples for the testing were prepared with industry-widespread unidirectional (UD) carbon fibres (PX35FBUD030 consisting of Panex 35 50K rovings with an areal weight of 300 g/m^2^; supplier: Zoltek Ltd., Nyergesújfalu, Hungary).

The two types of composites were manufactured with the same prepregging laminating method for both the virgin and recovered carbon reinforcements. The carbon fibre layers were impregnated individually, followed by a curing process in a Heraeus UT20 ventilated drying oven (Hanau, Germany). The curing was carried out for 1 h at 150 °C and then for 1 h at 180 °C. The fully cured carbon composite sheets were stacked (the UD-CF sheets were placed in a [0]_5_ layup order) and hot-pressed together at 160 °C for 15 min with a hydraulic pressure of 15 bars in a T30 temperable platen press (Metal Fluid Engineering s.r.l., Verdello Zingonia, Italy). To achieve a nominal composite thickness of 4 mm, we utilised 5 layers of prepregged reinforcements for the UD-CFRP composites and 3 layers for the nonwoven felt reinforced CFRP composites.

To assess the fibre content of the composites, we weighed the dry reinforcement layers alongside the mass of the crosslinked composite sample with an Ohaus E01140 precision weighing scale (Nänikon, Switzerland). Subsequently, we determined the fibre content of the samples as a mass percentage:

(1) $$Fibrecontent\left(mass\%\right)=\frac{m_{dry\;reinforcement}}{m_{composite}}\cdot 100$$

The average fibre content of the composite samples was 42 wt% in the case of the UD-CFRP composites, and 22 wt% in the case of the nonwoven felt reinforced composites.

The recycling process of the CFRP laminates was optimised in a systematic experimental investigation. The carbon reinforcements were recovered by soaking the composites in a mixture of xylene (Molar Chemicals Kft., Halásztelek, Hungary) and diethylenetriamine (DETA, Merck, Darmstadt, Germany) at elevated temperatures in a drying oven. Then, new composite sheets were fabricated with the recovered reinforcements and fresh vitrimer with the abovementioned method. The optimising experiments and the optimised and upscaled recycling process are explained in detail in Section 3.1.

Each of the four types of composite samples (comprising CFRPs with virgin and recovered nonwoven carbon felt and UD-CF reinforcements) underwent dynamic mechanical analysis (DMA) using a Q800 equipment (TA Instruments, New Castle, DE, USA) to determine the glass transition temperature (*T_g_*) and the temperature dependence of storage modulus (*E*′). The nominal size of the DMA samples was 60 × 10 × 4 mm (the support span was 50 mm), and a three-point bending setup was applied. The oscillation frequency was set to 1.00 Hz, the static load was 0.10 N, a 0.00 N minimum oscillation force was applied, and a 125% force track was used. The oscillation strain was 0.02 mm, and the examined temperature range was between 30 and 150 °C, while the heating rate was set to 3 °C/min. The *T_g_* values were determined as the peaks of the tan*δ* curves with TA Instruments’ Universal Analysis 2000 version 4.7A software.

The recycled carbon fibres and the fracture surfaces of vitrimer composites made from virgin and recycled carbon fibres were examined using a JEOL JSM 6380LA scanning electron microscope (SEM, JEOL Ltd., Tokyo, Japan). The samples were coated with gold using a JEOL JPC1200 cathodic sputtering gold plating apparatus. The acceleration voltage was between 5 and 15 V.

The virgin and recycled UD-CFRP composites were analysed by interlaminar shear tests on a Zwick Roell Z020 tensile testing machine (Ulm, Germany). The mechanical testing was carried out according to the ISO 14130:1997 standard short-beam method [24]. The sample size was 17.5 × 35 × 3.5 mm, and the support span was 17.5 mm. The testing speed was 1 mm/min, and 2 N preload was applied. The results were evaluated with the Zwick TestXpert 11.0 software; the interlaminar shear strength was calculated from the collected force data:

(2) $$\tau =\frac{3}{4}\cdot \frac{F}{b\;\cdot \;h}$$

where *τ* (MPa) denotes the interlaminar shear strength, *F* (N) denotes the maximum load or failure force value, *b* (mm) is the specimen width, and *h* (mm) is the specimen height. The significance of the recycling process’s effect on the interlaminar shear strength of the composites was investigated via a one-way analysis of variance (ANOVA) at a significance level of α = 0.05 with Minitab v17 software.

## 3. Results and Discussion

In this study, we first optimised the recycling process of the composites based on the recommendation of the vitrimer supplier. Once optimal parameters were identified, the recycling process was scaled up, accompanied by various mechanical and qualitative validation tests. The tests were performed on different carbon fibre reinforcements, including unidirectional and nonwoven felt, as the significant differences between the reinforcement structures may have led to different outcomes for the recycling and reprocessing experiments. The long-term goal of the recycling processes is to achieve the closed-loop recycling of the vitrimer matrix by transamination reactions, but the current study focused on the optimisation of carbon fibre recovery.

### 3.1. Optimisation of the Recycling Process

Previous data on recycling vitrimer composites indicated that the choice of organic solvents used during the fibre recovery process and the elevated temperature during the dissolving process could greatly impact recycling effectiveness. Our experiments revealed that not only do process parameters affect the quality of the recovered reinforcement, but also the technological steps of the process play a significant role.

#### 3.1.1. Optimal Parameters for the Recycling of Vitrimer Composites

We designed and conducted dissolving experiments to determine the optimal ratio of the mixture of organic solvents and dissolving temperature. We followed the supplier’s recommendation and utilised a mixture of xylene and DETA. DETA played the primary role in breaking the covalent bonds within the crosslinked polymer network by transamination reactions, while xylene was used as a diluent agent for economic and industry-applicability reasons. The solvent ratio and dissolving temperature were the factors of this two-round experiment. The effectiveness of the recycling was measured via the layers’ separation time and mass loss of the composite samples. The weighing of the recovered reinforcements was carried out after the evaporation of the solvent residue (the evaporating process happened in a Heraeus UT20 drying oven at 80 °C for 8 h). For the optimisation experiments, the three-layer nonwoven felt reinforced composites were used (the sample size was 15 × 15 × 4 mm), as well as a total of 100 mL solvent mixture (to avoid oversaturation).

Firstly, the optimal ratio of the DETA–xylene mixture was determined with room-temperature solvents. The results of the experiment can be seen in Table 1. The dissolving times of the different mixtures did not differ substantially; however, the ratio of DETA in the mixture crucially influenced the mass loss of the samples: the 20% DETA mixture caused a 56.98% mass loss, followed by the 10% DETA mixture with a 52.59% mass loss and the 30% DETA mixture with a 43.76% mass loss. Based on the matrix removal efficiency and economic reasons, we chose the 20:80 DETA–xylene mixing ratio as the optimum ratio.

Secondly, the effect of dissolving temperature on the recycling efficiency was examined in the case of the optimal solvent mixing ratio. The results of the experiment can be seen in Table 2. The increase in dissolving temperature affected both the matrix removal (measured via the samples’ mass loss) and the dissolving time substantially; therefore, it is recommended to carry out the recycling of vitrimer composites at an elevated temperature.

Based on the results of the optimisation experiments, we scaled up the recycling process and subjected approximately 200% larger composite samples by weight to further examination. We applied for the upscaling tests nonwoven carbon felts and unidirectional carbon fabrics. This allowed us to investigate the reproducibility of the process and the quality of the composites fabricated from the recovered reinforcements. The upscaled recycling process with the optimal parameters was as follows: the composite sheet was submerged in a DETA–xylene solvent mixture with a mixing ratio of 20:80, and the solvent mixture was approx. five times more by weight than the composite sample. The dissolving occurred in a well-sealable glass container (to prevent solvent evaporation during the process) at 80 °C in a drying oven. Two 4 h dissolving cycles were applied. The recovered reinforcements were rinsed with gentle rinsing motions in a xylene bath, and the carbon fibres were dried at room temperature for 40 h after each cycle; the last drying process was supplemented by elevated temperature drying for 8 h at 80 °C to evaporate the solvent residue from inside the reinforcement.

**Figure 1:**
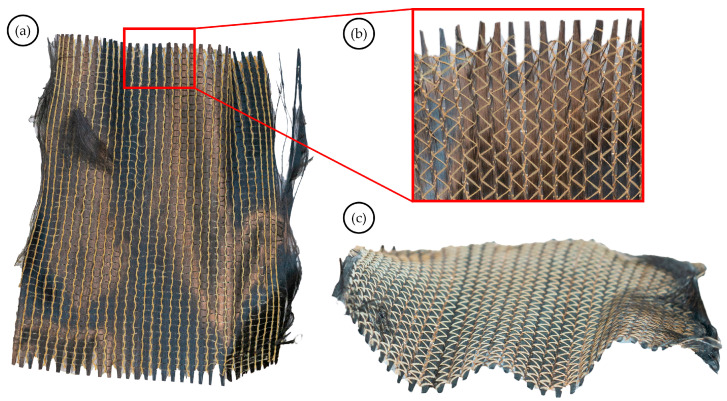
Images of the typical defects of the recovered reinforcements: (**a**) damaged edges of the fabric; (**b**) roving separation; (**c**) waviness of the dry and recovered fabric.

In addition to qualitative and quality-specific examinations, significant empirical insights regarding the recycling process can be drawn. During the dissolving process, the solvent solution can become oversaturated, which may inhibit the further dissolving of the vitrimer matrix; therefore, the frequent replacement of the solvents is needed (in this case, the solvents were replaced before the second dissolving cycle). The rinsing process before drying the reinforcements is mandatory; otherwise, the residue of the solvent–vitrimer mixture can re-solidify around the fibres, causing the reinforcement to become rigid and stiff. This makes handling the reinforcement challenging and may cause damage during subsequent reproduction processes. In the case of oriented, woven fibre reinforcements, the reinforcement structure could be damaged, as seen in Figure 1a. The fibres on the edges of the reinforcement layers can be tangled, which can be alleviated by trimming to maintain the expected fibre orientation in the reprocessed composite. Also, as seen in Figure 1b, the woven structure can loosen, and rovings can move apart from each other, which may weaken the mechanical properties of the reprocessed composite (compared to the composites made from virgin fibres) and can decrease the expected fibre content. During the drying phase of the recycling process, the reinforcements can become wavy due to the thermoformability of the residual vitrimer matrix, the inhomogeneous heat conduction, and the release of the recycle layers, as seen in Figure 1c. However, due to the vitrimers’ thermoformability, this defect can be corrected by hot-pressing the reclaimed reinforcements.

#### 3.1.2. SEM of Recovered Reinforcements

**Figure 2:**
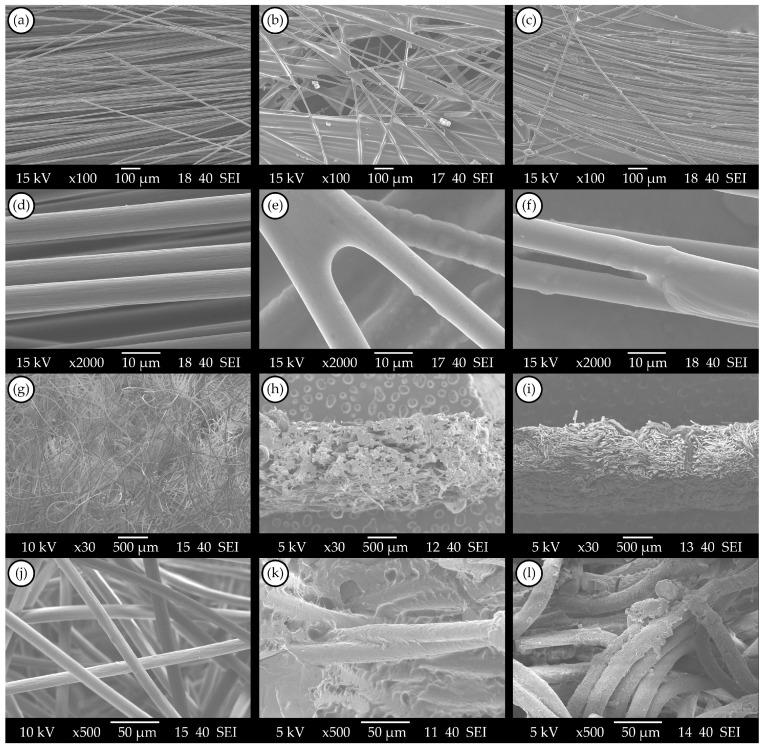
Scanning electron microscopy images: (**a**,**d**) structure and fibres of virgin unidirectional fabric; (**b**,**e**) recovered unidirectional carbon fibres after one dissolving cycle; (**c**,**f**) recovered unidirectional carbon fibres after two dissolving cycles; (**g**,**j**) structure and fibres of virgin nonwoven felt; (**h**,**k**) recovered nonwoven felt after one dissolving cycle; (**i**,**l**) recovered nonwoven felt after two dissolving cycles.

Scanning electron microscopic (SEM) images were taken to examine the amount of residual matrix inside the recovered reinforcement and to discover the possible adhesion properties of the carbon fibres. In the case of the UD fibres (Figure 2b), it can be clearly seen that a significant amount of matrix residue can be found after the first dissolving cycle; the residue forms a membrane-like structure, which connects the individual carbon fibres. Due to the connections, the reinforcement layer can behave as a semi-prepregged layer. This observation aligns with the empirical finding of the layers exhibiting rigid properties after the initial dissolving cycle. Figure 2e shows that matrix residue also remained around the individual fibres, which can reduce the adhesion quality in future reproduction processes when a different matrix material is applied to the recovered fibres. The arch-type formations between fibre crossings can result from the surface tension of the liquid solvent–vitrimer mixture, which re-solidified after solvent evaporation. The reinforcement after the second dissolving cycle (Figure 2c) better resembles the structure of the virgin carbon fibres; less matrix residue can be found between the individual fibres. These results also indicate the importance of solvent replacement and the rinsing process. However, based on the smooth surface quality of the recycled carbon fibres (Figure 2f) compared to the grooved surface structure of virgin fibres (Figure 2d), it can be stated that vitrimer residue can still be found on the carbon fibres’ surfaces after the optimal recovering process. In the case of nonwoven felt reinforcement, similar conclusions can be made. As Figure 2h,k show, a significant amount of matrix residue can be found after one dissolving cycle. Nevertheless, after the second dissolving cycle, the effectiveness of matrix removal was substantially better (see Figure 2i,l), which also evinces the importance of frequent solvent replacements and the rinsing process during the recycling of vitrimer matrix composites. The amount and form of the residual matrix apparently differ in the case of the two types of reinforcements: in the nonwoven felt, more residual matrix can be found, forming a less smooth surface around the carbon fibres. This may suggest that the nonwoven felt structure is harder to penetrate for the solvents, and the matrix residue cannot be removed as easily as in UD fabrics.

The carbon fibre reinforcements used were sized for an epoxy resin matrix, and the vitrimer is an epoxy-based polyimine, so strong adhesion, even chemical bonding can occur between the fibre and the matrix. Therefore, the complete removal of the matrix would require such a large excess of solvent and such a long time that the method would no longer be economically or environmentally viable. Based on the SEM images of the recovered reinforcements, it can be stated that with the proposed optimal recycling process, the majority of the matrix can be removed, which makes high-quality reprocessing possible. However, as vitrimer residue still can be found on the fibre surfaces, the recycling process should be further developed if we would like to apply the recycled fibres with different matrix materials during the reprocessing. In the case of the same matrix material, the residue may not be problematic due to the good self-adhesive properties of polyimine vitrimers.

### 3.2. Results of Validation Tests of Nonwoven CFRP Recycling

We produced composite sheets using recycled nonwoven felts and a virgin vitrimer matrix. Comparative SEM analysis and DMA were carried out to examine the reprocessability of the recovered fibres and to investigate the effect of the optimised recycling process on the mechanical properties of the reprocessed composite.

#### 3.2.1. SEM Images of the Nonwoven CFRP Composites

**Figure 3:**
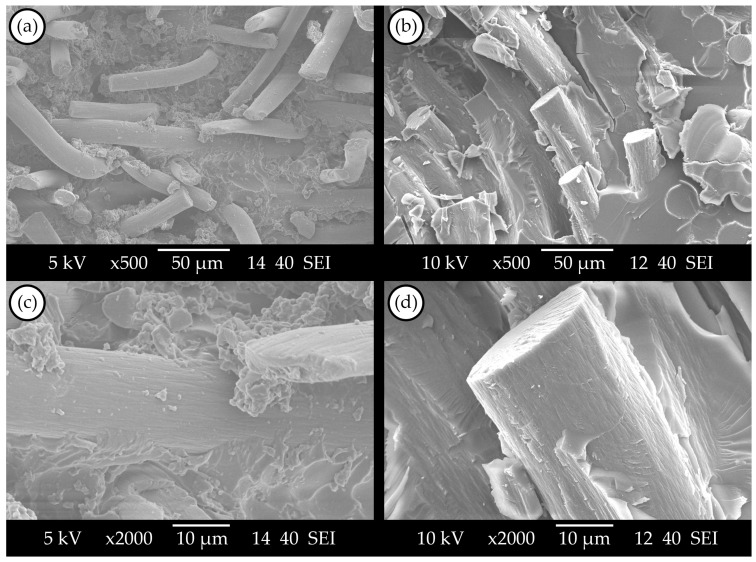
Scanning electron microscopy images: (**a**,**c**) virgin nonwoven felt CFRP; (**b**,**d**) recycled nonwoven felt CFRP.

SEM images were taken of a virgin nonwoven felt-reinforced CFRP composite and a reprocessed CFRP composite made with recovered nonwoven felt reinforcement to examine the influence of recycling on the impregnation and adhesion quality. The comparison of the virgin composite (Figure 3a) and the reprocessed composite (Figure 3b) shows that the impregnation quality is not significantly influenced by the recovering process of the fibres; a high degree of fibre embeddedness can be observed. The matrix’s rigid fracture surface in Figure 3c,d, along with the lack of fibre pull-outs and fibre–matrix separation, indicates strong adhesion in both composites. Based on the SEM images of the nonwoven felt CFRP composites, the proposed recycling process does not cause considerable defects in further reprocessing manufacturing.

#### 3.2.2. DMA Results of the Nonwoven CFRP Composites

**Figure 4:**
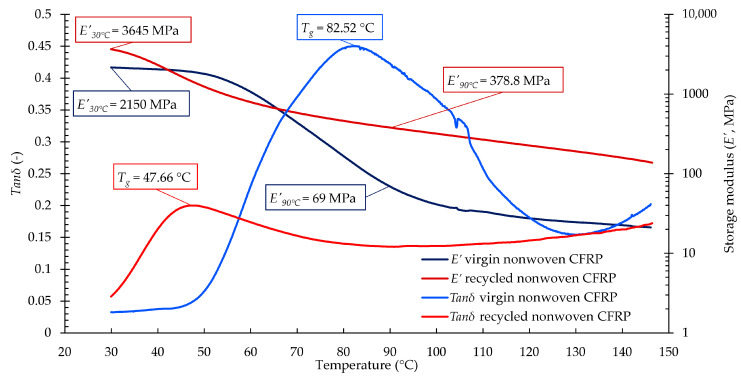
Dynamic mechanical analysis (DMA) results of virgin and recycled nonwoven felt CFRP composites.

We conducted a dynamic mechanical analysis on the composites made with recycled nonwoven felt to assess the effect of recycling on the mechanical properties. The results show that the glass transition temperature (*T_g_*) decreased by 42%, while the storage modulus (*E*′) values increased substantially (both at room temperature and above *T_g_*) as an effect of the fibre-recovering technology (see Figure 4). As the storage modulus indicates the material’s capability of storing energy elastically [25], the increase in *E*′ and the decrease in *T_g_* suggest that residual solvents and matrix may have been trapped inside the structure of the nonwoven felt (despite the extensive drying and evaporating processes). The remaining solvents may have interacted with the virgin vitrimer matrix during the impregnation and curing of reprocessed composite manufacturing. The DMA results of the nonwoven felt CFRP composites indicate that a significant change in mechanical properties should be expected when recovering fibres with the proposed method. The proposed recycling method should be further developed to provide a constant, well-designable quality of reprocessed composites. Due to drastic changes in glass transition temperature, no further mechanical analysis was performed with this composite type.

### 3.3. Results of Validation Tests of UD-CFRP Recycling

We produced composite sheets using recycled UD carbon fibres and a virgin vitrimer matrix. Comparative SEM analysis, DMA, and interlaminar shear testing were carried out to examine the reprocessability of the recovered fibres and to investigate the effect of the optimised recycling process on the mechanical properties of the reprocessed composite.

#### 3.3.1. SEM Images of the UD-CFRP Composites

**Figure 5:**
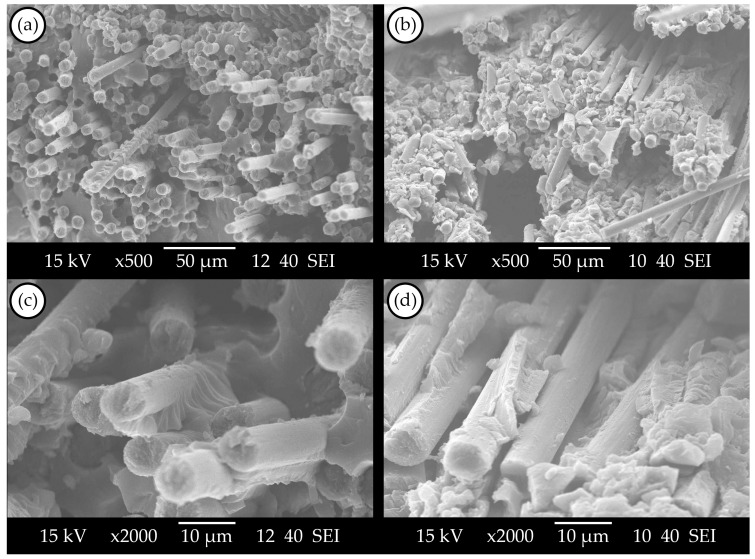
Scanning electron microscopy images: (**a**,**c**) virgin UD-CFRP composite; (**b**,**d**) recycled UD-CFRP composite.

SEM images of virgin and recycled UD-CFRP composites can be seen in Figure 5. Compared to the nonwoven felt CFRPs (Figure 3), more fibre pull-outs and also some voids can be seen in Figure 5a,b. The presence of residual vitrimer in recovered reinforcements might lead to an increase in void content. This residue could create small, closed cavities within the layers, hindering the penetration of the virgin matrix during impregnation in the reprocessing stage. However, as these defects can be found in both virgin and recovered fibre composites, it cannot be clearly stated that the proposed recycling process decreased the quality of the UD-CFRP. Also, in Figure 5c,d, good adhesion between the vitrimer matrix and carbon fibres can be observed on the fibre surfaces; therefore, the observable defects on the fracture surfaces (e.g., fibre pull-outs and voids) are not an effect of the recycling process. These defects typically occur in the case of UD reinforcement failures due to the orientation of the fibres. The random fibre orientation of the nonwoven felt made the fibre pull-outs less possible.

#### 3.3.2. DMA Results of the UD-CFRP Composites

**Figure 6:**
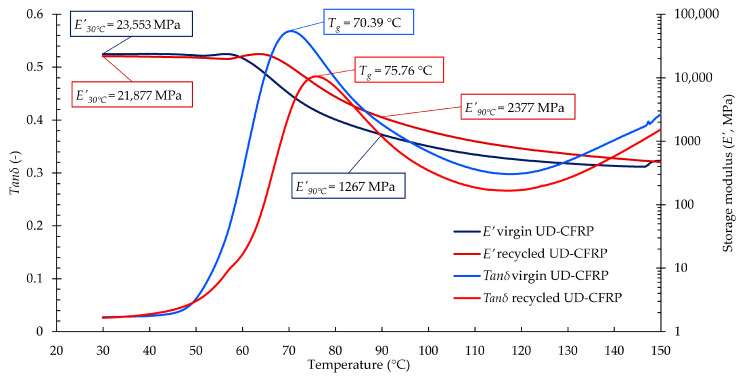
Dynamic mechanical analysis (DMA) results of virgin and recycled UD-CFRP composites.

The DMA results (Figure 6) give a better understanding of the changes in the mechanical properties of virgin and recycled UD-CFRPs. The *T_g_* of the recycled composite increased by 8%, although the *E’* value at room temperature decreased by 7%, which means that the reprocessed composite became slightly more flexible. Moreover, the *E’* value above *T_g_* was almost two times higher in the case of the reprocessed composite than in the case of the virgin UD-CFRP composite. The increase in the *T_g_* and increased *E’* above the *T_g_* suggest that the recycled composite has greater resistance to deformation at elevated temperatures, a phenomenon which needs further investigation. The literature has described the mechanical properties of recycled vitrimer matrixes and those of recycled fibres themselves. In the case of a recycled vitrimer matrix, a slight decrease is usually observed [17,18], while for the fibres, the mechanical properties typically remain within the same range [19,20]. Based on the DMA results of the UD-CFRPs, the proposed optimal recycling process may have no significant disadvantageous influence on the dynamic mechanical properties of the recycled composites; they may even be more resistant to heat-induced changes.

The notable variations in mechanical properties and thermal response observed in the DMA test could be attributed to the structural differences between the reinforcements utilised in the two composites. The random fibre orientation and the sponge-like structure of the nonwoven felt could have made the solvent penetration and the rinsing of the layers more difficult; thus, more residual matrix remained in the reinforcement. Also, the structure may have inhibited the total evaporation of the solvents, which could have resulted in significant changes.

#### 3.3.3. Interlaminar Shear Test Results of the UD-CFRP Composites

**Figure 7:**
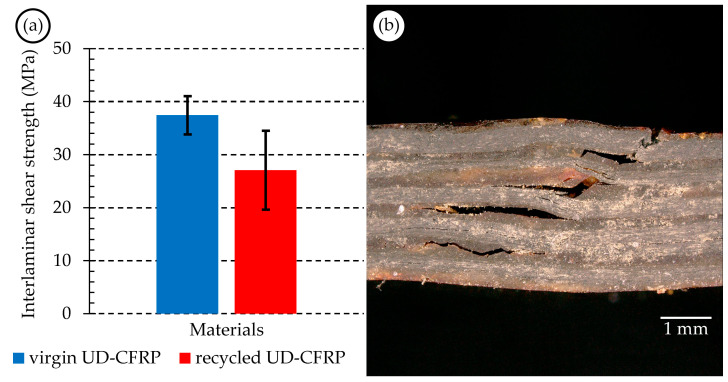
(**a**) Interlaminar shear test results of virgin and recycled UD-CFRP composites (error bar is two times the corrected sample standard deviation); (**b**) representative image of the specimens’ interlaminar shear failure mode (image taken with VHX-5000 digital optical microscope (Keyence, Mechelen, Belgium), lens: VH-Z20UT 20-200x).

Interlaminar shear tests, carried out by the short-beam method, were performed to better understand the adhesion quality in the case of the virgin and recycled UD-CFRPs, as residual solvent content or residual matrix content may have a negative effect on the adhesion capability of the virgin matrix. According to the ISO 14130:1997 standard [24], the failure of the samples is acceptable from the perspective of the shear mode failure (see Figure 7b). It can be seen in Figure 7a that the average interlaminar shear strength decreased by 27% (*τ*_virgin UD-CFRP_ = 37.45 MPa; *τ*_recycled UD-CFRP_ = 27.09 MPa), and the deviation of the interlaminar shear strength increased in the case of the reprocessed composites (the corrected sample standard deviation of *τ*_virgin UD-CFRP_ is 1.74 MPa; the corrected sample standard deviation of *τ*_recycled UD-CFRP_ is 2.50 MPa). This means that recycling the reinforcement causes deterioration in the mechanical properties and more uncertainty regarding production quality. The one-way ANOVA showed that the recycling process of the fibres significantly influences the interlaminar shear strength of the reprocessed composite (F-value = 0.000; *p*-value = 57.930) at a significance level of α = 0.05. Interlaminar shear failures are highly dependent on macroscopic material defects such as improper impregnation and void content. In accordance with the empirical quality inspection of the composites, some areas with inadequate impregnation can be observed, supporting the hypothesis of the adverse impact of voids caused by residual matrix presence. Further refinement of the proposed recycling process holds promise in mitigating the macroscopic defects of the reprocessed composites attributed to the residual matrix in the recovered reinforcement.

## 4. Conclusions

In this study, the recycling process of polyimine-type vitrimer based CFRP composites was optimised experimentally, and the upscaled process was tested with two different types of reinforcements: (i) unidirectional carbon fibre layers and (ii) nonwoven felts. Comparative material tests were carried out via dynamic mechanical analysis (DMA), interlaminar shear testing, and scanning electron microscopy (SEM) on the virgin and reprocessed composites to determine the effectiveness and quality of the recycling process. Based on this case study, the following conclusions can be drawn:

- Effective reinforcement recovery is feasible by dissolving the polyimine-type vitrimer matrix in a diethylenetriamine (DETA)–xylene organic solvent mixture with a mixing ratio of 20:80%. Elevated temperatures (in this case, 80 °C) decrease the dissolving time substantially.
- Empirical experience and SEM images showed that frequent solvent replacement and rinsing in xylene after the dissolving process are essential to minimise the amount of residual matrix.
- The SEM images of the virgin and reprocessed composites showed no fundamental differences between the compared composites, and good adhesion between the matrix and fibres can be observed. In the case of the UD-CFRPs, higher void content and more fibre pull-outs were found on the fracture surfaces.
- The DMA results showed that in the case of the nonwoven felt CFRPs, the recycling process decreased the glass transition temperature (*T_g_*) and increased the storage modulus (*E*′) values (giving the composite a more elastic property), which could be an effect of the residual solvent in the reinforcement. In the case of the UD-CFRPs, the reprocessed composite exhibits greater resistance to deformation at higher temperatures (higher *T_g_* and higher *E’* values above the glass transition temperature).
- Notable variance in the material properties of the recycled composite might arise from the impact of the reinforcement structure on solvent evaporation. Specifically, the nonwoven felt structure may retain more residual solvents during the reprocessing stage, potentially leading to interaction with the newly applied virgin matrix.
- The interlaminar shear tests showed that the reprocessed UD-CFRP composites had lower average interlaminar shear strength with a higher deviation as a possible result of the lower impregnation quality of the reprocessed composites.
- Based on the results of this study, the recycling of fibre-reinforced vitrimer composites is feasible and more straightforward than in the case of conventional thermoset matrix composites; however, the optimisation of the recycling processes is essential to make the recycling processes environmentally and economically sustainable so as to mitigate any potential adverse effects stemming from the residual matrix and solvents in the recovered reinforcement.

## Figures and Tables

**Table 1:** Effect of solvent ratios on the recycling of vitrimer composites.

DETA–xylene Ratio (%)	Sample Mass before Dissolving (g)	Sample Mass after Dissolving (g)	Dissolving Time (min)
10:90	0.6541	0.3101	400
20:80	0.6579	0.2830	390
30:70	0.6720	0.3779	360

**Table 2:** Effect of dissolving temperature on the recycling of vitrimer composites.

Temperature (°C)	Sample Mass before Dissolving (g)	Sample Mass after Dissolving (g)	Dissolving Time (min)
25	0.6673	0.2553	390
80	0.6735	0.2198	120

## Data Availability

The raw data supporting the conclusions of this article will be made available by the authors on request.
